# Effect of Post Weld Heat Treatment on the Microstructure and Electrochemical Characteristics of Dissimilar Material Welded by Butter Method

**DOI:** 10.3390/ma13204512

**Published:** 2020-10-12

**Authors:** Umer Masood Chaudry, Hafiz Waqar Ahmad, Muhammad Rehan Tariq, Ameeq Farooq, Muhammad Kashif Khan, Farooq Sher, Hassan Zeb, Kotiba Hamad

**Affiliations:** 1 School of Advanced Materials Science & Engineering, Sungkyunkwan University, Suwon 16419, Korea; umer@skku.edu; 2 School of mechanical Engineering, Sungkyunkwan University, Suwon 16419, Korea; 3 Department of Metallurgy and Materials Engineering, University of the Punjab, Lahore 54590, Pakistan; Rehan.tariq112@gmail.com (M.R.T.); ameeqfarooq.mme@pu.edu.pk (A.F.); 4 School of Chemical Engineering, Sungkyunkwan University, Suwon 16419, Korea; kashif.khan167@gmail.com; 5 SKKU Advanced Institute of Nanotechnology (SAINT), Sungkyunkwan University, Suwon 16419, Korea; 6 School of Mechanical, Aerospace and Automotive Engineering, Faculty of Engineering, Environmental and Computing, Coventry University, Coventry CV1 5FB, UK; 7 Institute of Energy & Environmental Engineering, University of the Punjab, Lahore 54590, Pakistan; hassaanzeb@hotmail.com

**Keywords:** Ni alloy 617, 12Cr, butter weld, post weld heat treatment, microstructure, potentiodynamic polarization, Arrhenius plot

## Abstract

In the present study, the effect of post weld heat treatment (PWHT) on the microstructure and corrosion kinetics of butter welded Nickel Alloy 617 and 12Cr steel was investigated. Buttering was carried out on the 12Cr side with the Thyssen 617 filler metal. Furthermore, post weld heat treatment (PWHT) was conducted at 730 °C with a holding time of 4 h followed by furnace cooling. Optical Microscopy (OM) was conducted to study the microstructural evolution in dissimilar material welding as a result of PWHT. Moreover, Scanning Electron Microscopy with energy dispersive spectroscopy (SEM-EDS) was employed to determine the elemental concentrations in all important regions of the butter weld before and after the PWHT. In addition, the effect of PWHT on the corrosion kinetics of the butter weld was also investigated by potentiodynamic polarization measurements in 5 wt.% NaCl + 0.5 wt.% CH_3_COOH electrolyte at room temperature, 30 °C, 50 °C and 70 °C. The corrosion activation parameters were also determined for both the samples by using Arrhenius plots. The results revealed the higher susceptibility of corrosion of the butter weld after PWHT, which was attributed to the reduced Cr content in the heat affected zone of the 12Cr region due to the sensitization effect of the heat treatment, resulting in higher corrosion rates.

## 1. Introduction

“Global warming is one of the most important issues mankind is currently facing. It is increasing day by day due to the burning of fossil fuels in very large amounts around the world to meet energy demands. Nowadays nearly 60% of the world’s power generation depends upon fossil fuels. Thermal power plants are burning coal in huge amounts resulting in the emission of flue gases like carbon dioxide (CO_2_), sulphur dioxide (SO_2_) and carbon monoxide (CO) leading to many ecological problems like air pollution and global warming. To reduce emission of these harmful gases in the environment has become the most important concern of many countries. The scientists and engineers are deploying various strategies to reduce concentration of the flue gases (CO_2_, SO_2_) by increasing the efficiency of the thermal power plants currently running or using other renewable resources of energy generation [1,2]. Therefore, solar and wind energies are some of the most common renewable energy resources adopted by many countries to meet their energy requirement. Moreover, immense research has been carried out on an advanced ultra-super critical thermal power plant that can withstand temperatures beyond 700 °C. Generally, a 0.5% increase in the power generation can be achieved by increasing the steam temperature by 10 °C [3,4,5,6]. Therefore, in order to improve the efficiency of thermal power plants, there is the need for an appropriate material for the manufacturing of turbine rotor and blades, which can be utilized at extreme conditions i.e., above 700 °C and 250 bar temperature and pressure, respectively.

For this purpose, Nickel based alloys of grades 617, 625 and 740 are considered to be the most promising materials owing to their good mechanical and metallurgical properties [7,8]. During steam turbine operation, the stage of low pressure and high temperature is considered to be most destructive for material prospect. Accordingly, a material with high fatigue strength and low corrosion kinetics at higher temperatures is required. 12Cr steel with its low cost and high corrosion resistance is considered to be the suitable material to serve this purpose. However, the bottleneck to use alloy 617 and 12Cr as a rotor material requires the designing of dissimilar material welding technology. Therefore, a reliable welding technology is required to fabricate hybrid structures to withstand the extreme conditions. Few researchers have already investigated the welding of alloy 617 with various materials and the consequent effects on the properties were evaluated. For instance, Hosseini et al. investigated the microstructure and weldability of Inconel 617 and 310 austenitic steel using Inconel 82, 617 and 310 austenitic steel as a filler materials [9]. The results revealed that Inconel 617/310 austenitic steel joints welded by using Inconel 617 filler metals demonstrated the highest solidification cracking resistance. While on the other hand, 310 steel filler joints showed the lowest cracking resistance, which was attributed to low melting point and secondary precipitate. Rando et al. studied the low cycle fatigue behavior of Inconel 617 weldments using gas tungsten arc welding (GTAW) at room temperature and at 800 °C in air [10]. The fatigue tests showed the decrease in fatigue life with increased total strain and temperature. The inferior fatigue characteristics were attributed to the accumulation of the plastic strain and ductility of material at elevated temperature. Nonetheless, no comprehensive literature is available to thoroughly access the microstructural and corrosion properties of dissimilar material welding between 12Cr and Alloy 617 after the PWHT. 

Accordingly, in this study, a dissimilar welding was carried out between Nickel Alloy 617 and 12Cr steel using buttering welding technique. The PWHT was carried out on the butter weld to relieve the residual stresses and its consequent effects on microstructure and corrosion kinetics were evaluated. 

## 2. Materials and Methods

**Figure 1:**
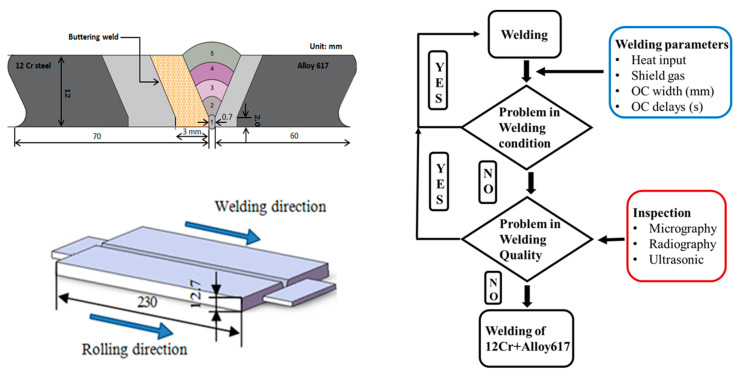
Buttering welding of Alloy 617 and 12Cr.

Chemical composition of all the materials used in the present study is shown in Table 1 [11]. Thyssen 617 was utilized as a filler metal for the multipass dissimilar and buttering weld. The schematics of butter welding in shown in Figure 1. The welding was performed with a total of 7 passes using gas tungsten arc welding (GTAW) welding technology under direct current straight polarity (DCSP) conditions. Before the dissimilar welding, butter welding was performed by Thyssen 617 filler metal on the 12Cr side. The surface was carefully observed after each welding pass for any deficiencies or defects such as porosity or incomplete fusion. Furthermore, the post weld heat treatment (PWHT) was carried out at the heating rate of 220 °C/min and sample was held at 730 °C for 4 h and then the furnace cooled. Optical microscopy (JEOL, Suwon, Korea) was used for the microstructural analysis of the important regions (Alloy 617, Alloy617-HAZ, weld metal, 12Cr-HAZ, 12Cr) of Ni617-12Cr butter weld. SEM-EDS was performed for evaluating the elemental distributions in all regions in the butter welded before and after the PWHT. For electrochemical analysis, a weldment coupled sample (containing Alloy 617, Alloy617-HAZ, Weld zone, 12 Cr-HAZ and 12 Cr base metal) of dimensions 10 mm × 10 mm × 10 mm was carefully cut from the butter weld. The samples were then mounted in the holder for conduction by exposing the 1 cm^2^ surface area and the remaining surface was insulated by using epoxy resin. A three-electrode cell coupled with a potentiostat (PAR 273A, Princeton, NJ, USA) was used for electrochemical characterization. Graphite electrode and saturated calomel electrodes (SCE) with saturated KCl as an electrolyte were used as counter and reference electrodes, respectively. The 5 wt.% sodium chloride (NaCl) + 0.5 wt.% glacial acetic acid (CH_3_COOH) according to national association of corrosion engineers (NACE) TM 0177 prepared in distilled water was used as an electrolyte. The band heater, along with the thermo controller and thermocouple was used to maintain the electrolyte temperature. The potentiodynamic polarization technique was used to measure the electrochemical properties like corrosion current density (*i_corr_*), dissolution rate and corrosion potential. During polarization testing, the samples were polarized cathodically to −250 mV from its open circuit potential or free potential with scan rate of 0.166 mV/s and then polarized anodically to +250 mV from its open circuit potential with the same scan rate. After the potentiodynamic polarization scan, the electrochemical parameters were calculated using PowerCORR^®^ corrosion measurement software (273A, Princeton applied research, Oak Ridge, TN, USA) and corrosion activation parameters using Arrhenius plots.

## 3. Results and Discussion

**Figure 2:**
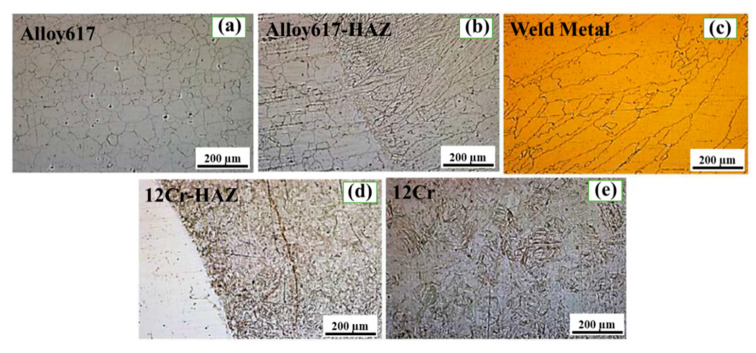
Optical micrographs of important regions before post weld heat treatment (PWHT): (**a**) Alloy 617, (**b**) Alloy617-HAZ, (**c**) Weld metal, (**d**) 12Cr-HAZ and (**e**) 12 Cr.

**Figure 3:**
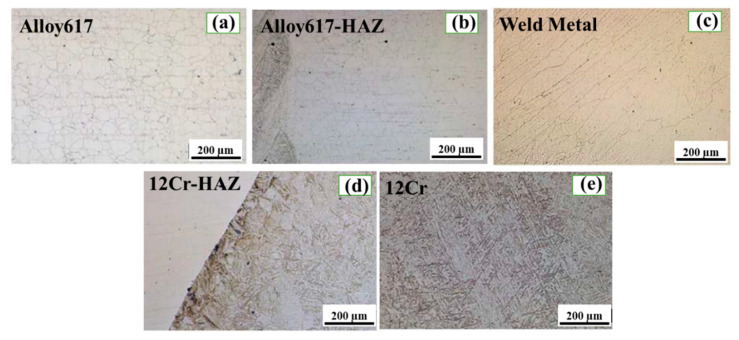
Optical micrographs of important regions after PWHT: (**a**) Alloy 617, (**b**) Alloy617-HAZ, (**c**) Weld metal, (**d**) 12Cr-HAZ and (**e**) 12 Cr.

To investigate the effect of PWHT on the microstructure evolution of the important constituents of the dissimilar butter weld, optical microscopy was carried out. Figure 2 and Figure 3 show the optical micrographs (OM) of all the regions of the dissimilar butter weld before and after the PWHT, respectively. It is evident from Figure 2a that Ni 617 consists of austenitic grains, which can also be observed in Figure 3a but with a less homogeneous microstructure. However, no significant effect of PWHT on Ni 617 was observed due to its excellent heat resistant characteristics. Moreover, the HAZ of Alloy 617 (before and after the PWHT in Figure 2b and Figure 3b, respectively) showed a sudden increase in the grain size, which can be attributed to the grain growth due to welding heat input. The micrograph of the weld metal (Figure 2c) confirmed the presence of the dendrite grains with the largest grain size compared to the other regions based on the highest tendency for grain growth due to maximum heat exposure. The dendritic microstructure suppresses the extreme segregation of alloying elements and impurities in the interdendritic regions, which can consequently lead to the enhanced strength and ductility of the weld metal [12]. The martensitic grains were observed in the 12Cr-HAZ before the PWHT (Figure 2d), and they were found to be deformed due to solid phase transformation by welding heat. The martensitic grains were detected in 12Cr for before and after the PWHT (Figure 2e and Figure 3e, respectively). Moreover, to determine the effect of PWHT on the elemental distribution in all the important regions of the dissimilar butter weld, SEM with EDS was performed on the weld before and after the PWHT. The SEM-EDS plots taken from the before and after PWHT butter welded samples line scan and the results are presented in Table 2. It can be noticed that no significant difference was observed in the compositions of major constituents (Alloy 617, 12Cr) as a result of PWHT. However, a slight decrease in the Ni concentration was detected in the weld metal compared to the base metal. This can be attributed to the use of Thyssen 617 as a filler metal, which is almost identical to Alloy 617 as can be seen from Table 1. Moreover, the buttering weld was found to consist of molybdenum (Mo), chromium (Cr), iron (Fe) and cobalt (Co), which was due the dilution of 12Cr and Thyssen 617 during the buttering weld process. Furthermore, no remarkable difference in the concentration of major elements (Fe, Ni and Mo) was noticed after the PWHT. However, the concentration of Cr was found to be decreased in the 12Cr-HAZ after the PWHT, which was due to the sensitization effect imparted by the PWHT [13]. 

**Figure 4:**
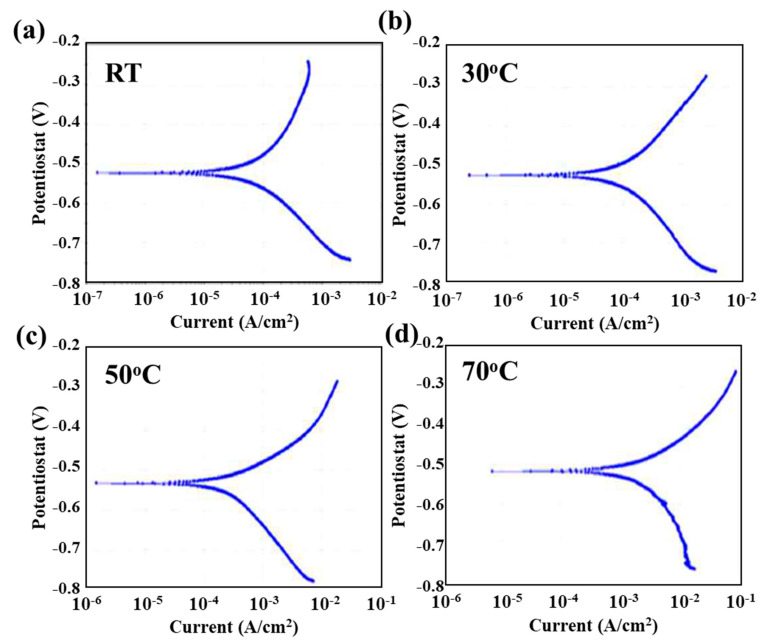
Potentiodynamic polarization curve of weldment couple before PWHT at (**a**) RT, (**b**) 30 °C, (**c**) 50 °C and (**d**) 70 °C.

**Figure 5:**
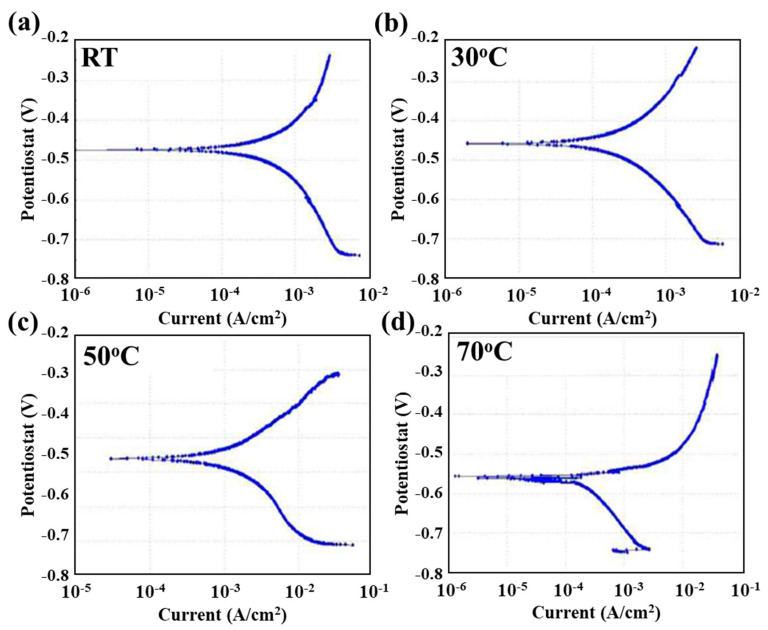
Potentiodynamic polarization curve of the weldment couple after PWHT at (**a**) RT, (**b**) 30 °C, (**c**) 50 °C and (**d**) 70 °C.

To determine the effect of PWHT on the electrochemical behavior of the weldment couple sample, the sample before and after the PWHT were immersed in 5 wt.% NaCl + 0.5 wt.% CH_3_COOH electrolyte at different temperatures (RT, 30 °C, 50 °C and 70 °C). Figure 4 and Figure 5 show the potentiodynamic polarization curves for the weldment couple before and after PWHT at various temperatures, respectively. The Tafel extrapolation approach was used to calculate the corrosion potential (*E_corr_*) and corrosion current density (*i_corr_*) while corrosion rate was calculated using formula [14] as shown in Equation (1). The values of kinetic parameters corrosion rates for both samples at various temperatures are illustrated in Table 3. The electrochemical properties of base alloys (alloy 617 and 12Cr) also illustrated in Table 3 for comparison purpose. It can be seen from the Table 3 that alloy 617 showed low corrosion rates at various temperatures as compared to the 12Cr owing to its excellent corrosion resistant. The corrosion rate of both base alloys increased with the increase in temperature.

(1) $$Corrosion\;rate\;(mm/y)=\frac{(3.27\times 10^{-3})\times i_{corr}\times EW}{\rho }$$

where ‘*EW*’ is the equivalent weight of the alloy in grams (g), ‘*ρ*’ is the density of alloy in grams per centimeter cubed (g∙cm^−3^) and ‘*i_corr_*’ is the corrosion current density in ampere per centimeter squared (A∙cm^−2^). E.W. can be calculated by the following equation:

(2) $$E.W.\left(\mathrm{equivalent}\;\mathrm{weight}\right)=\frac{1}{\sum \frac{n_{{\mathrm{if}}_{i}}}{W_{i}}}$$

here W_i_, f_i_ and n_i_ are the atomic weight, weight fraction and valence of the ith element in the alloy.

The purpose to conduct the potentiodynamic polarization test in corrosive electrolyte of 5 wt.% NaCl solution (brine) of concentration up to saturation level and buffer acidification through 0.5 wt.% CH_3_COOH at different temperature was used to simulate the service conditions. The potentiodynamic polarization scans with anodic branches and cathodic branches shifted towards the high current density value with the increase in the temperature while the corrosion potential (*E_corr_*) value almost remain same as shown in Figure 4 and Figure 5. Even the PWHT also does not affect the *E_corr_* value as there was a slight shift in the value when compared to that recorded at room temperature (RT). However, when we compare the results after PWHT (Figure 5) with the increase in temperature from RT to 70 °C, the value of *E_corr_* shifted towards a more active potential. The hydrogen reduction was occurred at the cathodic polarization branch due to the de-aerated acidic condition of the electrolyte according to the following reaction [15]:

2H^+^ (aq) + 2e^−^ → H_2_ (g) (3)

The evolution of hydrogen gas bubbles occurred on the sample surface during cathodic polarization. In the anodic polarization region, there was no sign of passivation or formation of any barrier film as all the curves show the rapid dissolution behavior. The nickel and chromium metals show the passive behavior in the acidic electrolyte but due to the presence of the high concentration of Cl^−^ ions which act as a de-polarizer and weldment couple which act as a galvanic couple. The anodic polarization from Figure 4 and Figure 5 show that the dissolution was due to the Fe which was present in the weldment couple portion of 12Cr steel in large wt.% according to the following reaction [15]:

Fe (s) → Fe^+2^(aq) +2 e^−^ (4)

The corrosion rates were increased with the increase in temperature for both the samples (before and after PWHT). The corrosion rate of the weldment couple before heat treatment was measured to be 0.0121 mm/y at RT, which was increased to 0.0164 mm/y at the solution temperature of 30 °C. Moreover, the corrosion rate was remarkably increased to 0.0924 mm/y (~660 % higher compared to RT) when the solution temperature was maintained at 70 °C. It can be attributed to the fact that when the temperature is increased, the pH of the electrolyte shifted towards more an acidic value and the mobility of the anion species, i.e., Cl^−^ ions, increases. The Cl^−^ ions are a strong oxidizing agent and cause pitting or localized corrosion in passive alloys like nickel alloys; they penetrate through the porous oxide film and can be absorbed on surface resulting in higher deterioration of the surface of those alloys [16,17,18,19]. After absorbing on the surface, these ions can promote the diffusion of metal ions from the surface to the corrosive medium leading to formation of metal hydrides [20]. As in Figure 4 at 70 °C, the cathodic curve shows the diffusion control behavior on the sample surface during the reduction process [21,22]. Moreover, it can be seen from Table 3 that the weldment after the PWHT demonstrated an identical trend, as the corrosion rates increased with the increase in solution temperature (0.0156 and 0.1037 at RT and 70 °C, respectively). However, the samples with PWHT showed higher corrosion rates compared to the samples without PWHT in similar testing conditions. This may be attributed to the reason that PWHT led to the sensitization of Cr, which reduced the concentration of Cr in the 12Cr-HAZ and 12Cr region (Table 2), resulting in high corrosion susceptibility. Although the temperature effect is evident, the basic polarization curve remained the same, indicating that the temperature only heavily effected the corrosion rate not the corrosion mechanism. 

Furthermore, the activation energy (*E_a_*) was calculated to estimate the adsorption behavior of Cl^−^ ions on the surface of the weldment couple before and after the PWHT. The dependence of corrosion rate on the temperature was determined using the following Arrhenius equation [23]:

(5) $$i_{corr}=Ae^{\frac{-E_{a}}{RT}}$$

where ‘*A*’ is the pre-exponential factor, ‘*E_a_*’ is the activation energy for surface dissolution in joules per mole (J mol^−1^), ‘*R*’ is the universal gas constant whose value is 8.314 J mol^−1^ K^−1^, ‘*T*’ is the absolute temperature in kelvin (K) and ‘*i_corr_*’ is the corrosion current density in amperes per centimeter squared (A cm^−2^). The value of *E_a_* for both the samples was calculated from the slope of the plot between ln *i_corr_* and the inverse of the absolute temperature (1/*T*). Moreover, transition state theory was utilized to calculate the enthalpy (Δ*H*) and the entropy (Δ*S*) from the following equation of the corrosion rate process [22]:

(6) $$i_{corr}=\frac{RT}{Nh}e^{\frac{\Delta S}{R}}e^{\frac{\Delta H}{RT}}$$

**Figure 6:**
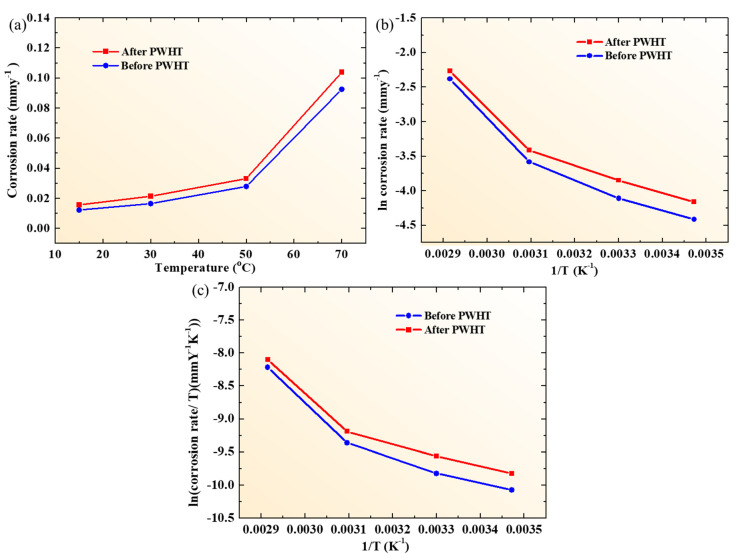
(**a**) Variation in corrosion rate with respect to solution temperature for weldment couple before and after the PWHT, (**b**) and (**c**) are the Arrhenius plot and ln (*υ_corr_*/T) vs. 1/T for corrosion of weldment couple before and after the PWHT, respectively.

Where ‘*N*’ is the Avogadro’s number whose value is 6.023 × 10^23^ mole^−1^, ‘*h’* is the Planck’s constant whose value is 6.626 × 10^−32^ Js. The values for Δ*H* and Δ*S* can be calculated from the slope (−Δ*S*/*R*) and intercept (ln (*R*/*Nh*) + Δ*S*/*R*) from the graph between (ln *i_corr_*/*T*) and inverse of absolute temperature (1/T). Figure 6a–c shows the various plots for calculating the activation parameters for the corrosion process of both the samples and results are summarized in Table 4. Figure 6a presents the variation of the corrosion rate as a function of temperature and it is evident that the corrosion rate is in direct relation with the temperature of the corrosion solution. Figure 6b illustrates the Arrhenius plot and the *E_a_* values were calculated to be 35.34 ± 1.51 kJ/mol and 32.62 ± 1.85 kJ/mol for the weldment couple before and after the PWHT, respectively. As it is well understood that the *E_a_* is the resistance offered by the material to the initiation of corrosion so lower activation energy reflects the lower resistance for metal corrosion. In addition, after the formation of the passive layer, the corrosion process will be further pushed to the regions of higher *E_a_* values, indicating that corrosion of the alloy will occur on the uncovered areas which will lower *E_a_* [22]. Accordingly, it implies that the corrosion is thermodynamically more convenient for the weldment after the PWHT rather than before the heat treatment. In addition, the entropy of activation (Δ*S*) is negative for both the samples, which can be attributed to the reason that the activated complexes in the rate determining step need lower energy for completion of the reaction. It can be seen from Table 4 that the weldment couple after the PWHT has lower Δ*S* values compared to the sample without the heat treatment, which reflects the high kinetics for the corrosion reaction. 

## 4. Conclusions

The effect of PWHT on the microstructural evolution and corrosion characteristics of butter welded Ni617-12Cr alloy was studied. The OM analysis confirmed the absence of any significant change in the microstructure of all the important regions after the PWHT. The SEM-EDS analysis revealed the reduction of Cr content in the 12Cr-HAZ and the 12Cr alloy region after the PWHT. Moreover, the corrosion characteristics showed that the PWHT lead to the increase in corrosion kinetics of the butter welded Ni617-12Cr, which was attributed to the occurrence of sensitization as a result of heat treatment. Moreover, the calculation of enthalpy of activation and entropy of activation also confirmed the same trend. Furthermore, it is pertinent to mention that, based on the current study, the dissimilar material welding between alloy 617 and 12Cr steel seems to be suitable; so, accordingly, more efforts are required to extend the current welding process to industrial scale. 

## Figures and Tables

**Table 1:** Chemical composition of Alloy 617, Thyssen 617 and 12 Cr steel. Data from [11].

Base/Filler Metal	Chemical Composition (Weight %)											
	Ni	Cr	Co	Mo	Al	C	Fe	Si	Ti	Cu	Mn	S
**Alloy 617**	44.3	22	12.5	9.0	1.2	0.07	1.5	0.5	0.3	0.2	0.5	0.008
**Thyssen 617**	45.7	21.5	11.0	9.0	1.0	0.05	1.0	0.1	1	-	-	-
**12Cr**	0.43	11.6	-	0.04	-	0.13	Bal.	0.4	-	0.1	0.58	-

**Table 2:** EDS elemental characterization of materials before and after PWHT.

Elements	Mo (wt.%)		Cr (wt.%)		Fe (wt.%)		Co (wt.%)		Ni (wt.%)	
Condition	B-PWHT	A-PWHT	B-PWHT	A-PWHT	B-PWHT	A-PWHT	B-PWHT	A-PWHT	B-PWHT	A-PWHT
**Alloy 617**	10.98	10.87	22.03	22.26	-	-	12.47	12.43	54.07	54.03
**Alloy617-HAZ**	9.77	10.15	20.25	21.19	2.29	2.48	11.95	11.84	53.17	53.08
**Weld Metal**	10.88	10.56	20.31	20.83	3.00	3.11	12.31	12.46	51.25	51.86
**Butter Welded metal**	8.71	8.94	21.59	21.39	13.13	13.25	10.68	10.71	45.89	45.53
**12Cr-HAZ**	1.36	1.27	11.80	10.56	85.56	86.69	-	-	-	-
**12Cr**	-	-	11.84	10.28	86.93	88.43	-	-	-	-

**Table 3:** Effect of PWHT on the corrosion characteristics at various temperatures.

Material	Alloy 617	12Cr Steel	Corrosion Properties before PWHT		Corrosion Properties after PWHT	
Temperature (°C)	Corrosion Rate (mm/y)	Corrosion Rate (mm/y)	*i_corr_* (µA/cm^2^)	Corrosion Rate (mm/y)	*i_corr_* (µA/cm^2^)	Corrosion Rate (mm/y)
RT(15)	0.0089	0.0238	1.214	0.0121	1.55	0.0156
30	0.0104	0.0238	1.6536	0.0164	2.143	0.0213
50	0.0147	0.0398	2.789	0.0278	3.311	0.0330
70	0.0193	0.1776	9.2693	0.0924	10.40	0.1037

**Table 4:** Activation parameters for butter weld samples before and after the PWHT.

Material	Activation Energy, *E_a_* (kJ mol^−1^)	Enthalpy, Δ*H* (kJ mol^−1^)	Entropy, Δ*S* (J mol^−^^1^ K^−^^1^)
Before PWHT	35.34 ± 1.51	31.95 ± 2.79	−125.31 ± 3.44
After PWHT	32.62 ± 1.85	25.45 ± 2.24	−172.55 ± 3.93
